# The Washington Code of e-Health ethics and other ethical codes

**DOI:** 10.2196/jmir.2.suppl2.e3

**Published:** 2000-09-13

**Authors:** Ahmad Risk

**Affiliations:** ^1^Internet Healthcare CoalitionUSA

## Abstract

The goal of the e-Health Code of Ethics is to ensure that people worldwide can confidently and with full understanding of known risks realise the potential of the Internet in managing their own health and the health of those in their care. The final e-Health Code of Ethics, presented in this paper, has been prepared as a result of the "e-Health Ethics Summit," which convened in Washington DC on 31 January 2000 - 2 February 2000. The summit, organized by the Internet Healthcare Coalition and hosted by the World Health Organisation/Pan-American Health Organisation (WHO/PAHO), was attended by a panel of about 50 invited experts from all over the world and produced the foundation for a draft code, which was released 18 February for an online public consultation period which ended on 14 April 2000. The final Washington e-Health Code of Ethics sets forth guiding principles under eight main headings: candor; honesty; quality; informed consent; privacy; professionalism in online health care; responsible partnering; and accountability.

**Full paper published as:**

Rippen H, Risk A, for the e-Health Ethics Initiative. e-Health Code of Ethics. Journal of Medical Internet Research 2000;2(2):e9. http://www.jmir.org/2000/2/e9/)

